# Image-guided stent-directed irreversible electroporation for circumferential ablation in the rat esophagus

**DOI:** 10.3389/fbioe.2022.934858

**Published:** 2022-11-17

**Authors:** Song Hee Kim, Seung Jeong, Jeon Min Kang, Yubeen Park, Dae Sung Ryu, Dong-Sung Won, Ji Won Kim, Chu Hui Zeng, Hyung-Sik Kim, Hong Bae Kim, Sang Soo Lee, Jung-Hoon Park

**Affiliations:** ^1^ Biomedical Engineering Research Center, Asan Institute for Life Sciences, Asan Medical Center, Seoul, South Korea; ^2^ Department of Gastroenterology, Asan Medical Center, University of Ulsan College of Medicine, Seoul, South Korea; ^3^ Department of Biosystems and Biomaterials Science and Engineering, Seoul National University, Seoul, South Korea; ^4^ Department of Mechatronics Engineering, School of ICT Convergence Engineering, College of Science and Technology, Konkuk University, Chungju-si, South Korea

**Keywords:** irreversible electroporation, self-expandable stent, esophagus, electrode, ablation

## Abstract

**Background:** Irreversible electroporation (IRE) has been investigated in the alimentary tract; however, the lack of dedicated electrodes and insufficient tissue responses made its application limited. The aim of this study was to investigate the efficacy and safety of image-guided stent-directed IRE in the rat esophagus.

**Methods:** The bipolar self-expandable electrode (SE) was developed using the braiding technique. A finite element analysis was performed to validate optimal electrical field strength for the rat esophagus. A total of 40 out of 50 rats received stent-directed IRE and were sacrificed at 10 h, 3 days, 7 days, and 28 days of 10 each. The remaining ten rats underwent a sham procedure. The outcomes of stent-directed IRE were assessed by esophagography and histological responses.

**Results:** Stent-directed IRE was technically successful in all rats with mild muscle contraction. The heart rate dropped immediately and gradually recovered at 180 s. TUNEL and caspase-3 with submucosal thickness significantly increased at 10 h and Day 3 compared with those of the sham control (all *p* < 0.001). The thickness of epithelial layers with collagen deposition significantly decreased at 10 h and Day 3 (all *p* < 0.001), however, increased at Day 7 compared with that of the sham control (all *p* < 0.05). The Ki67-positive deposition significantly increased at Day 3 and 7 compared with that of the sham control (all *p* < 0.001). All variables were similar to those of the sham control at Day 28.

**Conclusion:** Image-guided stent-directed IRE was effective and safe in the rat esophagus. It seems to have effectively and evenly induced cell death and gradually recovered with cellular regeneration.

## Introduction

Image-guided ablation modalities including radiofrequency ablation and irreversible electroporation (IRE) have been widely used to treat solid malignancies (Hong and Georgiades, 2010; Shah et al., 2013; Scheffer et al., 2014; Padia et al., 2016; Collettini et al., 2019). IRE is a local tissue ablation technology that uses short- and high-voltage electric pulses to cause cell death and has established as an alternative to thermal ablation for unresectable tumors, particularly those near critical vascular structures (Rubinsky, 2007; Rubinsky et al., 2007; Bulvik et al., 2016; Kotnik et al., 2019; Geboers et al., 2020; Batista Napotnik et al., 2021). IRE is well known as lower thermal damage, sparing the surrounding structures such as blood vessels or nerves, thus resulting in more rapid regeneration of the tissues (Scheffer et al., 2014; Padia et al., 2016; Collettini et al., 2019; Geboers et al., 2020). IRE applications with promising clinical outcomes are expanding in various organs such as the liver, pancreas, prostate, kidneys, and lungs (Rubinsky, 2007; Rubinsky et al., 2007; Collettini et al., 2019). Furthermore, the efficacy of endoluminal IRE was investigated in the alimentary tract including the esophagus, stomach, small bowel, and colon (Phillips et al., 2012; Luo et al., 2016; Jeon et al., 2021). Previous studies have outlined several limitations, such as the use of an electrode that is not suitable for the alimentary tract and lacks the proper IRE dose range and IRE-induced serial histological evaluation (Phillips et al., 2012; Luo et al., 2016; Jeon et al., 2021). The possibility of IRE to preserve the extracellular matrix, blood vessels, and nerve bundles in the rabbit esophagus was demonstrated. Non-thermal IRE in the esophageal tissue provided a rapid recovery of the IRE-treated esophagus at a 16-week follow-up study (Song et al., 2021). However, the effects of IRE on the esophagus still have limitations and have not been definitively elucidated yet.

Endoluminal ablation techniques with the use of catheters or balloon-based electrodes have been investigated to treat malignant obstructive disorders (Sharma et al., 2007; Steel et al., 2011; Auriemma et al., 2019; Inoue et al., 2020). However, if the electrode is not in sufficient contact with the target tissues, caused by severe anatomical curvature or relatively larger inner diameter ductal lesions, the therapeutic effects may be insignificant. Therefore, we hypothesized that a newly developed self-expandable electrode could provide a minimal invasive approach under fluoroscopic guidance and enhance the IRE-induced therapeutic effects by fully attaching the electrode to the inner wall of the alimentary tract while minimizing the IRE-induced thermal effect due to the crossed structure of nitinol wires. Thus, the purpose of this study was to investigate the efficacy and safety of the image-guided stent-directed IRE ablation with serial histological response in a rat esophageal model.

## Materials and methods

### Preparation of a self-expandable electrode

**FIGURE 1:**
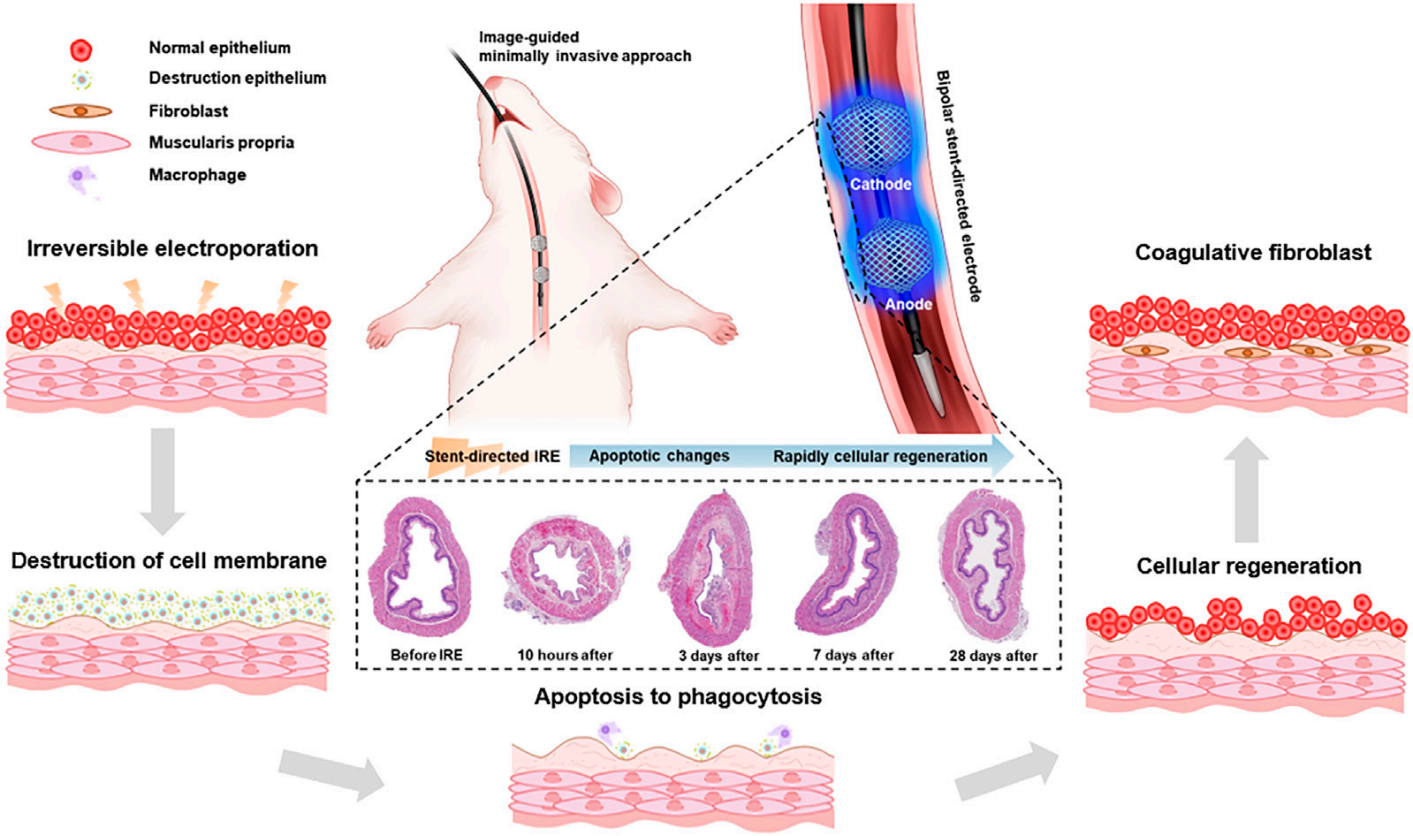
Schematic illustration of the stent-directed irreversible electroporation (IRE) system. The newly developed stent-directed IRE system was proven capable of providing a minimally invasive image-guided approach and enhances the IRE-induced therapeutic effects by fully attaching the electrode to the inner wall of the esophagus. The microscopic images show the serial histological changes over time.

A novel bipolar self-expandable electrode (SE) for IRE was developed to allow electric energy delivery to the rat esophagus (S&G Biotech Co., Ltd., Yongin, Korea) (Figure 1). The SE was modified using a plasma etching system (S&G Biotech Co., Ltd.) to improve electrical conductivity and remove micro-cracks affecting its durability. The surface characteristics of the SE were analyzed *via* scanning electron microscopy (SEM; AIS 1800C, Seron Technologies Inc., Uiwang, Korea). The bipolar SE consisted of two SEs with a pair of anode and cathode connected at a distance of 13 mm for separating insulation. Each SE was braided using 32 nitinol wires with a thickness of 0.09 mm. When fully expanded, the SE was 5 mm in diameter and 3 mm in length. Both ends of the SEs were fixed to the delivery system for recapture and removal after IRE. The delivery system was 8 Fr in diameter and 50 cm in usable length and comprised an insulating outer sheath and a pusher catheter with a guiding olive tip. The bipolar SE was connected to the IRE system (EPO-S1, The Standard Co., Ltd., Gunpo, Korea), which is capable of setting an electric field intensity up to 3 kV at pulse widths of 100–1,000 µs(with 100–2,000 µs pulse intervals).

### Potentiodynamic behavior

The anodic potentiodynamic polarization test was used to evaluate the corrosion potential and corrosion rate to examine the corrosion resistance of electrodes (Cemazar et al., 2018). The electrochemical cell consisted of a saturated calomel reference electrode (SCE), a pure platinum counter electrode, and a working electrode. The working electrode was encapsulated with a silicone sealant so that only 5 mm of the electrode length and diameter was exposed, in a deaerated 0.9% sodium chloride solution. Before the polarization test, the working electrode was stabilized for 10 min to reach equilibrium with no net current flow to the metal surface. Potentiodynamic polarization tests were performed using computer-controlled voltammetry (Portable BiPotentiostat/Galvanostat stat 400, Metrohm DropSens, Spain) with a scan rate of 1 mV/s.

### Simulation of electrical field strength and irreversible electroporation-induced thermal effects

A finite element analysis was performed using COMSOL Multiphysics 5.6 (Stockholm, Sweden) to simulate the electrical field strength and thermal distribution of bipolar SE geometry at 400 V, 600 V, and 800 V with 100 µs width, 2,000 µs interval, and 90 pulses between electrodes. The rat esophagus was generated with a diameter of 5 mm and a wall thickness of 0.5 mm. The electrical and thermal properties of the stimulated esophagus were determined according to the tissue property database. The esophageal region near the SE was formed with a denser mesh to evaluate more accurate analysis. The bipolar SE geometry was generated for the simulation. A stationary study solver was adopted for the electric field strength evaluation, and a time-dependent study solver was used for the thermal distribution evaluation around the bipolar SE in the stimulated rat esophageal model.

### Animal study design

This study was approved by the Institutional Animal Care and Use Committee of our institution and conformed to the US National Institutes of Health guidelines for humane handling of laboratory animals. A total of 50 Sprague–Dawley rats (Orient Bio, Seongnam, Korea), weighing 300–350 g at 9 weeks of age, were used. A total of 40 rats underwent stent-directed IRE ablation and were randomly selected to be sacrificed at 10 h (*n* = 10), 3 days (*n* = 10), 7 days (*n* = 10), and 28 days (*n* = 10) after the procedure by administering inhalable pure carbon dioxide. The remaining 10 age-matched healthy rats underwent the sham procedure. All rats were supplied with food and water *ad libitum* at 24 ± 2°C with a 12-h day–night cycle. The body weight of the rats was measured weekly until they were sacrificed.

### Stent-directed irreversible electroporation ablation

Anesthesia was induced by an intramuscular injection of 50 mg/kg zolazepam, 50 mg/kg tiletamine (Zoletil 50; Virbac, Carros, France), and 10 mg/kg xylazine (Rompun; Bayer HealthCare, Leverkusen, Germany). A micro-guidewire (Transcend; Boston Scientific, Watertown, Mass) was inserted through the mouth and negotiated into the stomach under fluoroscopic guidance, and an SE delivery system was advanced over the guidewire into the middle portion of the esophagus. The bipolar SE was expanded at the level of the mid-thoracic esophagus. The parameters for performing IRE were set as follows: electric field strength: 250 V/cm, pulse width: 100 µs, pulse interval: 2,000 µs, and pulse number: 90 pulses. The fully expanded SEs were recaptured into the delivery system and then removed.

### Electrocardiographic examination

The electrocardiography (ECG) signals were monitored by the Ag/AgCl electrodes (2223H, 3M, MN, United States) connected with an ECG amplifier (PSL-iECG, PhysioLab, Busan, Korea). ECG including the heart rate was continuously monitored and recorded at −90 s to 210 s to investigate the cardiac safety during stent-directed IRE ablation. Muscle contraction was also monitored during the IRE procedure.

### Esophagographic examination

All rats underwent esophagography using a contrast medium (Omnipaque 300; GE Healthcare, Shanghai, China) immediately after the procedure and immediately before their sacrifice to verify the luminal patency and esophageal perforation. The IRE-treated esophagus was divided into the anode, middle, and cathode segments. The luminal diameters were measured in all rats at the three segments of the IRE-treated rat esophagus using Photoshop software (version 6.0; Adobe Systems, Palo Alto, Calif). Measurements were repeated three times at each level, yielding an average value per level; these values were subsequently averaged to obtain an overall average diameter of the segment.

### Histologic examination

The surgical exploration of the esophagus and stomach was performed in all rats. The extracted tissue samples were immediately fixed in a 10% neutral buffered formalin for 24 h. The IRE-treated esophagus was transversely sectioned, and the sectioned esophageal tissues were analyzed by dividing into three segments: anode, middle between the two electrodes, and cathode regions. The slides were stained with hematoxylin and eosin (H&E) and Masson’s trichrome (MT). Histological evaluation using H&E staining determining the degree of inflammatory cell infiltration, the thickness of the epithelial layer and submucosa, and the cross-sectional submucosa areas was performed. The degree of collagen deposition was determined using MT-stained sections. The degrees of inflammatory cell infiltration and collagen deposition were subjectively determined according to the distribution and density of the cells, i.e., graded as 1 = mild, 2 = mild to moderate, 3 = moderate, 4 = moderate to severe, and 5 = severe. All scan of staining samples was performed using a digital slide scanner (Pannoramic 250 FLASH III, 3D HISTECH Ltd., Budapest, Hungary). Measurements were obtained using a digital microscope viewer (CaseViewer, 3D HISTECH Ltd.).

### Immunohistochemical examination

Immunohistochemistry was performed on the paraffin-embedded sections using terminal deoxynucleotidyl transferase-mediated dUTP nick and labeling (TUNEL; Millipore Co., MA, United States), caspase-3 (LifeSpan BioSciences Inc., WA, United States), and heat shock protein 70 (HSP70; LifeSpan BioSciences Inc.) as primary antibodies. The degrees of TUNEL, caspase-3, and HSP70-positive deposition were subjectively determined (1 = mild, 2 = mild to moderate, 3 = moderate, 4 = moderate to severe, and 5 = severe).

### Immunofluorescence examination

Immunofluorescence (IF) staining was performed using Ki-67 (1:200, SolA15, Thermo Fisher Scientific, MA, United States) antibody. Secondary antibody staining was performed using Alexa Fluor 594 (1:5,000, Thermo Fisher Scientific) and 4′-6-diamidino-2-phenylindole (DAPI, Invitrogen, CA, United States). The number of positive staining cells was subjectively determined according to the distribution and density of the cells. Average values for the number of positive staining cells were calculated using the following formula: 100 × (the number of positive cell/the number of total cell).

### Statistical analysis

Data are expressed as the mean ± standard deviation (SD). The differences between the groups were analyzed using the Mann–Whitney *U*-test, as appropriate. A *p*-value < 0.05 was considered statistically significant. Statistical analyses were performed using SPSS (version 27.0; IBM Corp., Armonk, NY, United States).

## Results

### Characteristics of a self-expandable electrode

**FIGURE 2:**
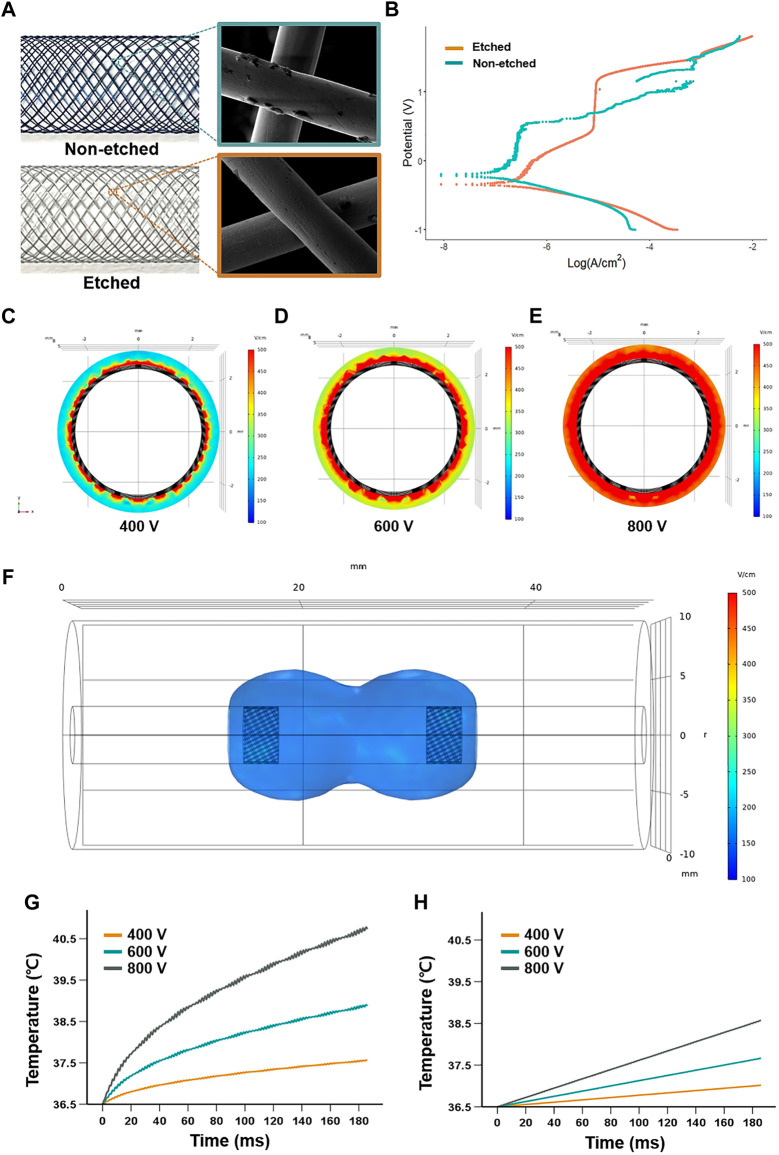
Characteristics and stimulation of the self-expandable electrode (SE). **(A)** SEM analysis of the surface characteristics of the nitinol wires before and after the plasma etching. **(B)** Anodic polarization curves of the electrical properties of the etched electrode, using 0.9% sodium chloride solution. The electric field distribution of axial-sectional views at the middle of the cathode when applied **(C)** 400 V, **(D)** 600 V, and **(E)** 800 V show a depth penetration of 0.204 mm, 0.315 mm, and 0.374 mm in a simulated esophageal model, respectively. **(F)** Representative iso-surface of the electric field at 400 V was applied. Graphs show that temperature changes over time at **(G)** the SE and **(H)** the middle portion between the two SEs when applied 400, 600, and 800 V.

The SE was successfully manufactured by the braiding technique using multiple nitinol wires. The analysis of SEM images after the plasma etching process morphologically revealed that the cracks on the surface of nitinol wires were significantly decreased (Figure 2A). The electrical properties of the etched SE were characterized by the potentiodynamic polarization. The anodic polarization curves for the tests conducted in 0.9% sodium chloride solution are shown in Figure 2B. The corrosion potential for the non-etched and etched SEs was 204 and 338 mVSCE, respectively. The passive behavior was stable for the etched SE and unstable for the non-etched SE, showing metastable pitting behavior in the passive regime. The passive current was approximately 10^−7^ A/cm^2^ for the non-etched SE and 10^−5^ A/cm^2^ when the transpassive potentials were above 1.0 VSCE. The interactions of the cathodic and anodic Tafel curves were extrapolated from the cathodic and anodic potentiodynamic polarization curves and used to determine the anodic current density (Icorr). The electric field distribution in the axially sectioned view revealed that the simulated depth penetrations were 0.204 mm at 400 V, 0.315 mm at 600 V, and 0.374 mm at 800 V applied voltage (Figures 2C–E). The shape of the iso-surface of the electric field at the longitudinal direction showed a dumbbell-shaped structure between cathode and anode SEs (Figure 2F). The temperature increased from a body temperature of 36.5°C and followed the logarithmic shape at the margin of the electrode, finally reaching 40.8°C (4.3°C increase) when the 800 V of the 90th pulsing ended, 38.8°C (2.3°C increase) for 600 V, and 37.6°C (1.1°C increase) for 400 V. At the midpoint between the SEs, the temperature increased linearly to 38.6°C (2.1°C increase) for 800 V, 37.7°C (2.2°C increase) for 600 V, and 37°C (0.5°C increase) for 400 V (Figures 2G,H). According to the depth penetration and temperature, the applied voltage of 400 V was determined for the *in vivo* rat esophagus.

### Procedural outcomes of stent-directed irreversible electroporation ablation

**FIGURE 3:**
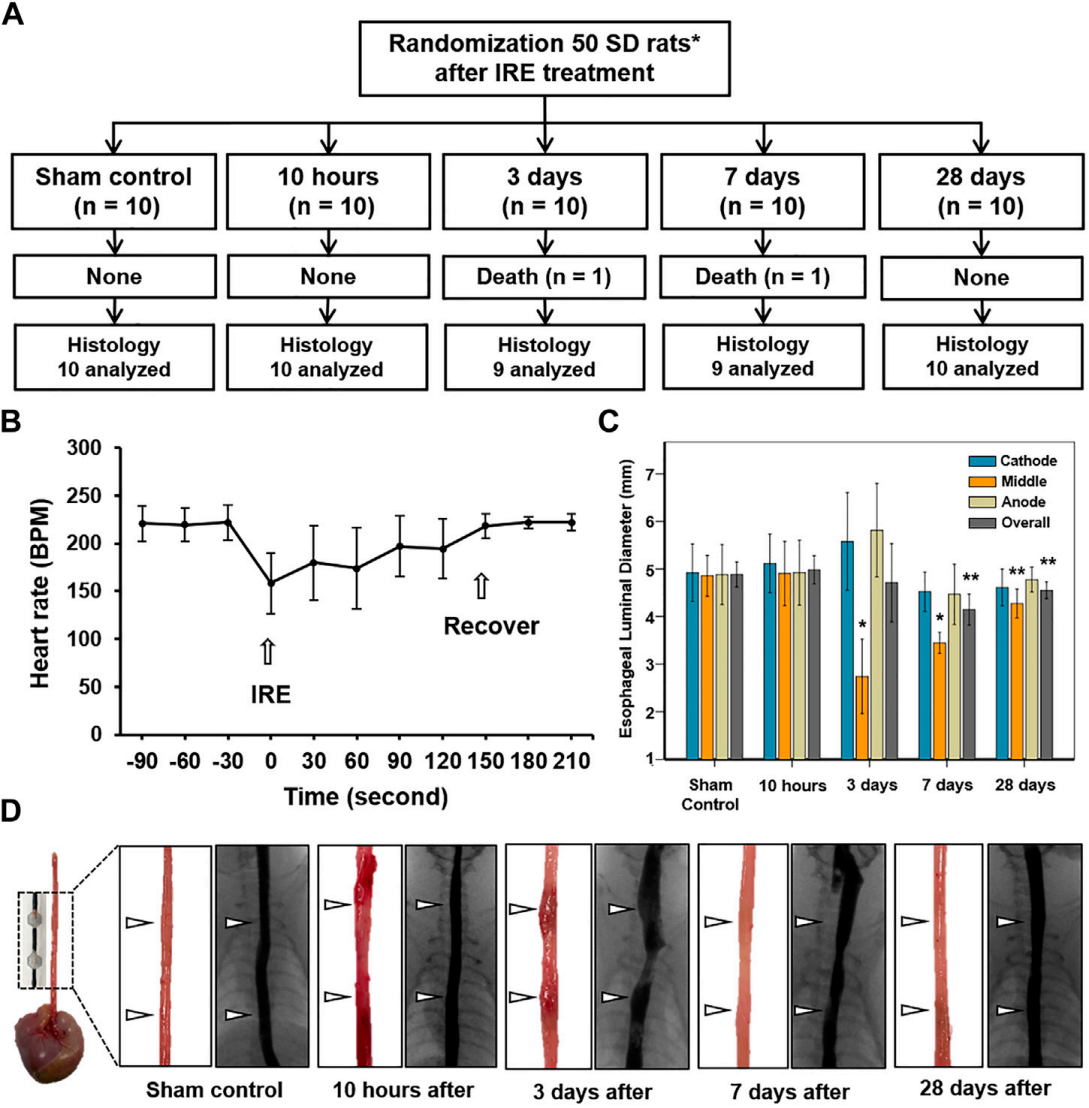
*In vivo* long-term experimental findings by IRE treatment. **(A)** Experimental flowchart showing the randomization process and study follow-up. **(B)** Graph shows that the electrocardiographic changes in the IRE-treated rats before and after the procedure. **(C)** Esophageal luminal diameter changes in study groups after IRE treatment. **(D)** Representative photographs of the gross and esophagographic images obtained from the sham control at 10 h, 3 days, 7 days, and 28 days after IRE treatment. Note: Data are presented as the mean ± SD. *p < 0.001 and **p < 0.05. IRE, irreversible electroporation.

Stent-directed IRE ablation under fluoroscopic guidance was technically successful in all rats without procedure-related complications. However, 2 (4%) of the 50 rats died during the procedure, owing to dyspnea due to a thick delivery system and were excluded from the analysis. The remaining 48 (96%) rats survived until the end of the study (Figure 3A). Upon application of IRE, mild muscle contraction was observed in all rats. The mean (±SD) heart rate was rapidly dropped from 221 ± 19 to 158 ± 32 beats per minute (BPM) immediately after IRE and gradually recovered to 222 ± 9 BPM at 180 s after IRE (Figure 3B). Although the body weights of the rats decreased at the first week after the procedure, this did not significantly affect any of these animals in terms of their general condition, behavior, and amount of food intake. The body weights gradually increased until their sacrifice.

### Esophagographic findings

The esophagographic findings are shown in Figures 3C,D. The mean (±SD) overall esophageal diameter did not differ at 10 h (4.98 ± 0.59 mm) and Day 3 (4.71 ± 1.66 mm) after IRE, but it became significantly smaller on Day 7 (4.14 ± 0.65 mm, *p* = 0.001) and Day 28 (4.55 ± 0.36, *p* = 0.035), compared with that of the sham control (4.89 ± 0.58 mm). The mean (±SD) diameter of the middle portion in between the two SEs at 10 h (4.91 ± 0.65 mm) was similar to that of the sham control (4.86 ± 0.47 mm, *p* = 0.876). However, the diameter dramatically decreased to 2.74 ± 0.74 mm (*p* < 0.001) on Day 3 but gradually recovered on days 7 (3.44 ± 0.21 mm, *p* < 0.001) and 28 (4.27 ± 0.29 mm, *p* = 0.020) despite statistical differences remained the same. Although esophageal narrowing was observed at Day 3 and 7 dysphagia symptoms such as poor food intake, nausea, and vomiting did not occur during the follow-up.

### Histological findings

**FIGURE 4:**
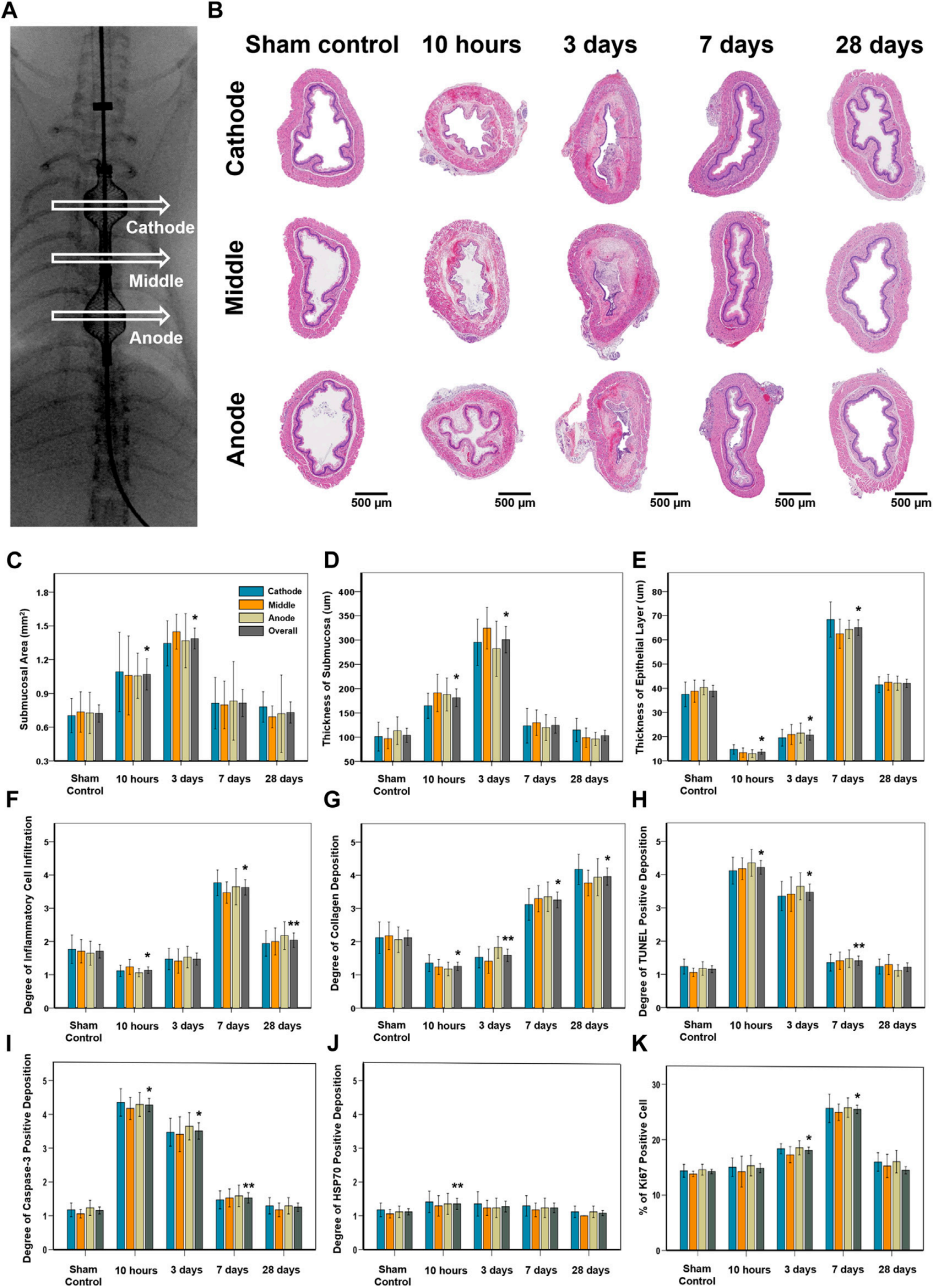
Histopathological findings in study groups after stent-directed IRE in the rat esophagus. **(A)** Radiographic image shows the location of the tissue sampled for histologic examination where the self-expandable electrode (SE) was present in the cathode SE, middle portion between the two SEs, and anode SE. **(B)** Representative hematoxylin and eosin-stained images show the axially sectioned esophagus after stent-directed IRE ablation showing serial histological changes. Histopathologic results of the sham control at 10 h, 3 days, 7 days, and 28 days after IRE treatment in **(C)** the submucosal area, **(D)** thickness of submucosa, **(E)** thickness of the epithelial layer, **(F)** degree of inflammatory cell infiltration, **(G)** degree of collagen deposition, degrees of **(H)** TUNEL, **(I)** caspase-3, and **(J)** HSP70-positive deposition, and **(K)** percentage of Ki67-positive cell. Note: Data are presented as the mean ± SD. *p < 0.001 and **p < 0.05.

**FIGURE 5:**
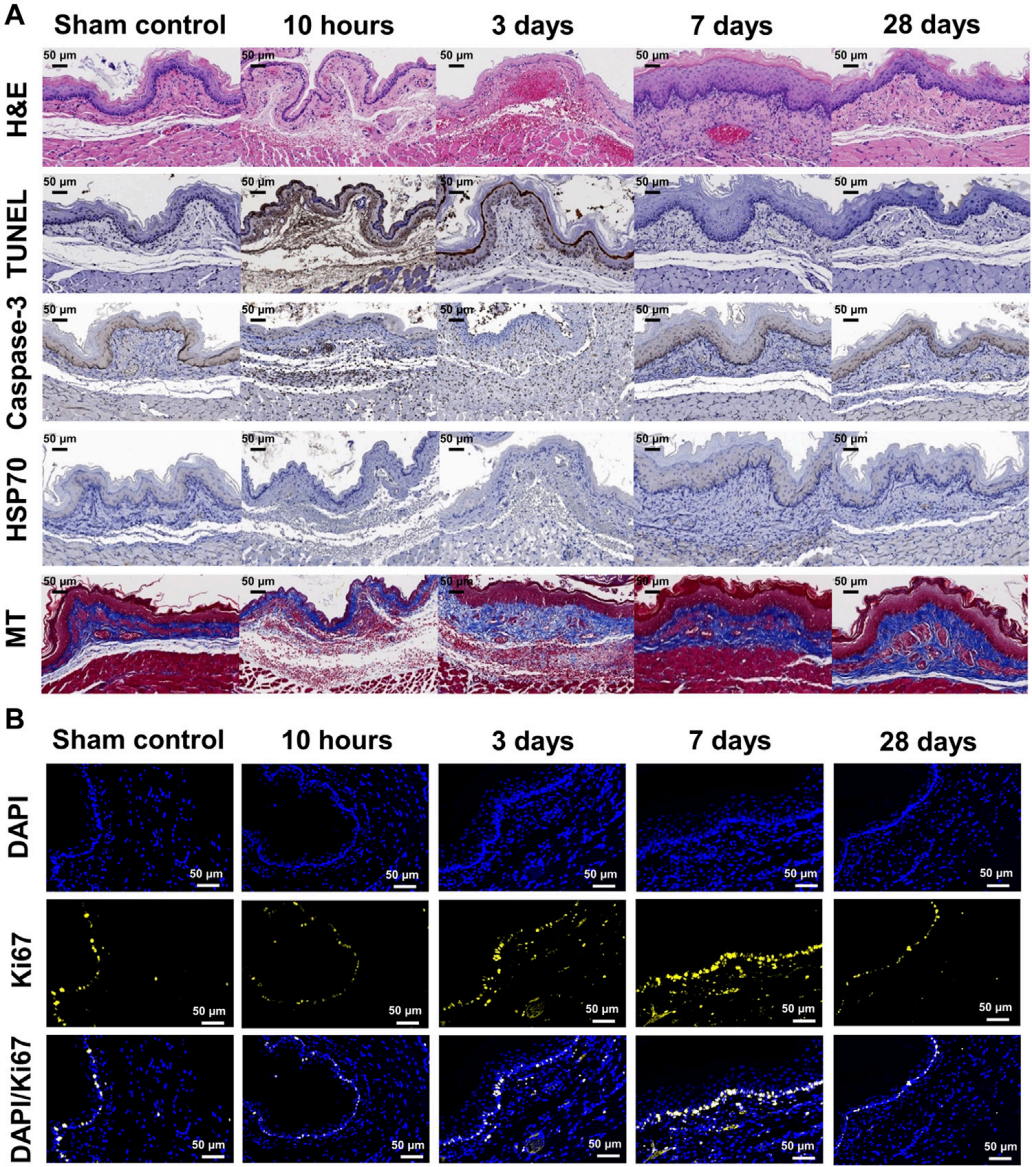
Representative microscopic images of histopathological sections. **(A)** Hematoxylin and eosin-stained, TUNEL, caspase-3, HSP70, and Masson’s trichrome-stained sections are shown (×20 magnification). Relative TUNEL and caspase-3 expression after stent-directed IRE increased at 10 h and 3 days after IRE treatment. Collagen deposition increased at days 7 and 28 . **(B)** Representative Ki67 immunofluorescence (DAPI, blue and Ki67, yellow) images showing active cellular regeneration at 7 days after IRE treatment (×40 magnification). Representative microscopic images of the histopathological sections of the adjacent organs of the IRE-treated esophagus.

The entire esophagus and stomach of all rats were successfully extracted. There were no serious IRE-induced complications such as bleeding or perforation in any of the enrolled study rats (Figure 3D). Histologic findings are summarized in Table 1, and examples are shown in Figures 4, 5. The overall submucosal areas and submucosal thickness were significantly increased at 10 h and Day 3 after IRE compared with those of the sham group (all *p* < 0.001). These changes were gradually decreased at Day 7 and similar to those of the sham group were observed at Day 28 after IRE treatment. The thickness of the epithelial layers was significantly decreased at 10 h and Day 3 after IRE treatment when compared with that of the sham group (all *p* < 0.001). The thickness of the epithelial layers was significantly increased at Day 7 compared with that of the sham group (*p* < 0.001). This difference was reduced to a level similar to that of the sham control at Day 28. The mean degree of inflammatory cell nitration did not differ at 10 h and Day 3 after IRE treatment, but it was significantly increased at Day 7 (*p* < 0.001) and Day 28 (*p* = 0.027) compared with that of the sham group (Figures 4A,B). The degrees of overall collagen deposition were significantly decreased at 10 h (*p* < 0.001) and Day 3 (*p* = 0.001) after IRE treatment; however, it was significantly increased at Day 7 and 28 compared with that of the sham control (all *p* < 0.001).

**TABLE 1:** Histological findings after stent-directed IRE ablation in the rat esophagus.

	Sham control	10 h	*p*-value	3 days	*p*-value	7 days	*p*-value	28 days	*p*-value
Submucosal area (mm^2^)	0.72 ± 0.15	1.07 ± 0.28	<0.001	1.39 ± 0.19	<0.001	0.81 ± 0.24	0.178	0.73 ± 0.17	0.874
Thickness of submucosa (µm)	103.9 ± 51.2	181.3 ± 64.3	<0.001	300.7 ± 96.1	<0.001	124.4 ± 57.1	0.059	104.2 ± 41.5	0.968
Thickness of the epithelial layer (µm)	38.87 ± 8.33	13.68 ± 3.56	<0.001	20.61 ± 7.49	<0.001	65.07 ± 11.45	<0.001	42.02 ± 6.06	0.061
Degree of inflammatory cell infiltration	1.71 ± 0.73	1.14 ± 0.35	<0.001	1.47 ± 0.64	0.087	3.63 ± 0.82	<0.001	2.04 ± 0.77	0.027
Degree of collagen deposition	2.12 ± 0.82	1.25 ± 0.44	<0.001	1.59 ± 0.67	0.001	3.25 ± 0.85	<0.001	3.96 ± 0.92	<0.001
Degree of TUNEL deposition	1.16 ± 0.37	4.22 ± 0.73	<0.001	3.47 ± 0.88	<0.001	1.41 ± 0.49	0.004	1.22 ± 0.46	0.478
Degree of caspase-3 deposition	1.17 ± 0.31	4.27 ± 0.69	<0.001	3.51 ± 0.73	<0.001	1.49 ± 0.54	<0.001	1.19 ± 0.41	0.450
Degree of HSP70 deposition	1.12 ± 0.33	1.35 ± 0.59	0.015	1.27 ± 0.59	0.091	1.24 ± 0.51	0.171	1.08 ± 0.27	0.510
Percentage of Ki67 cell (%)	14.26 ± 0.62	14.85 ± 1.31	0.176	18.07 ± 0.91	<0.001	25.48 ± 1.19	<0.001	14.49 ± 1.01	0.516

Note. Data are presented as means ± standard deviations.

### Immunohistochemistry findings

The degrees of overall TUNEL and caspase-3-positive depositions were significantly increased at 10 h and Day 3 after IRE treatment (all *p* < 0.001), and this difference was maintained at Day 7 compared with that of the sham group (*p* = 0.004 for TUNEL and *p* < 0.001 for caspase-3). These changes were decreased at Day 28, similar to those of the sham group. The degree of overall HSP70-positive deposition was slightly increased at 10 h after IRE treatment (*p* = 0.015), and there were no statistical differences at Day 3, 7, and 28 compared with the sham group.

### Immunofluorescence findings

The percentages of Ki67-positive deposition showed no significant difference at 10 h and Day 28 after IRE treatment. However, there were significantly increased at Day 3 and 7 compared with those of the sham group (all *p* < 0.001) (Figure 5B). These cells were localized to the area adjacent to the epithelial layer and maximal proliferating cell numbers were detected at Day 7 after IRE treatment.

## Discussion

The SE was able to contact the inner wall of the rat esophagus evenly, thereby enabling uniform energy delivery to the esophageal wall to maximize the IRE therapeutic effects. Stent-directed IRE ablation successfully and evenly ablated the submucosal tissues including epithelial layers of the rat esophagus. Well-distributed mucosal injuries were observed in the acute term and gradually recovered in similar to the sham group within Day 28. TUNEL and caspase-3 expression, which was activated in apoptotic cells, increased within highly ablated tissues at 10 h and Day 3 after IRE. Specifically, the ablated tissue maintained the preservation of basal cells in the mucosa layer and circular and longitudinal muscular cells at 10 h and Day 3 after IRE. The vessels remained structurally intact even in the region of the lamina propria which was severely ablated and fully recovered at Day 7. Basal cells in the stratified epithelium were found to be diverse cells at Day 7 after IRE compared to those of the sham group, indicating tissue homeostasis in which cells actively regenerate after IRE. Ki67 levels were intensified at Day 3 and 7 after IRE and were similar to those of the sham control at Day 28 after IRE.

Abnormalities of ECG signals are often reported as a representative side effect of various IRE procedures (Deodhar et al., 2011; Thomson et al., 2011; Aras and Efimov, 2018). In this study, the rat esophagus was ablated with the applied electric field strength at 250 V/cm, and the IRE-ablated rat esophagus was more than 5.2 mm away from the heart. The transient bradycardia occurred immediately after IRE, but the heart rate rapidly recovered to the baseline at 180 s after IRE. Although the IRE-treated rat esophagus was close to the heart, the low applied voltage did not induce consistent abnormalities in the rat heart. Our results support that sufficient electrical stimulation with a low voltage was possible in the rat esophagus with a relatively thin wall, unlike solid organs. This translational research on stent-directed IRE is considered to be sufficiently clinically applicable with cardiac safety.

IRE is reported to be a non-thermal tumor ablation technique that has been advocated to protect non-target tissues from thermal injury compared with other ablation modalities (Akahane et al., 2005; Al-Sakere et al., 2007; Onik et al., 2007; Garcia et al., 2011; Thomson et al., 2011). Although thermal damage may occur in the immediate vicinity of electrodes, especially when excessive high voltages are delivered (Garcia et al., 2014), IRE is mostly a non-thermal technique that can preserve the adjacent cellular structures with vessels (Weiss and Wolfgang, 2013). The potential thermal effects of the bipolar SE in the simulated results were evaluated. HSP70 expression in the IRE-treated rat esophagus, which is an indicator of heat stress (Al-Sakere et al., 2007), slightly increased immediately after IRE but did not differ between the sham control and IRE-treated groups. Stent-directed IRE with no thermal effects can be conducted with a low electric energy along with the structural characteristics using crossed nitinol wires of the SE.

Stent-directed IRE ablation could allow its clinical application as a palliative therapeutic option to endoluminal malignancies after all other treatment options are exhausted. The depth penetration and ablated area of the target lesion were easily controlled according to the applied electric field strength. The optimal ablation area was calculated using the simulation system before the procedure. Stent-directed IRE ablation has the advantage that the cellular regeneration of the surrounding tissues can be expected. The results of the present study provide evidence that stent-directed IRE ablation under fluoroscopic guidance is technically feasible and safe. Moreover, the SE made by multiple nitinol wire braiding may be sufficiently applicable to endoluminal RFA. The SE with self-expanding properties and high flexibility could be useful in the curved lesion.

There are several limitations to this study. First, the normal esophageal tissue differs from the malignant esophageal structure; hence, the efficacy of IRE for tumor treatment has not been sufficiently elucidated. Second, only a few representative markers of apoptosis and regeneration were evaluated in this study. Third, the IRE procedure necessitates neuro-muscular and cardiac continuous monitoring to observe the muscle stimulation and cardiac arrhythmias; however, in this study, continuous measurement was difficult in small animals. Fourth, given the disparity between human and rodent, the practical therapeutic process cannot be fully evaluated in this study and requires further investigation in large animal models. Fifth, the conductivity increases in the modeling of electric field distribution due to IRE and due to temperature increase (Corovic et al., 2013; Agnass et al., 2020) have not been sufficiently elucidated.

For future translational research studies, additional studies are needed to investigate the safety and efficacy of the stent-directed IRE system and to establish optimal electrical conditions on large animals; however, in the current study, the basic concept of stent-directed IRE ablation was verified as a novel endoluminal ablation modality. In conclusion, image-guided stent-directed IRE was effective and safe in the rat esophagus. It seems to have effectively and evenly induced cell death and gradually recovered with cellular regeneration.

## Data Availability

The original contributions presented in the study are included in the article/Supplementary Material; further inquiries can be directed to the corresponding author.
